# 5-HTTLPR modulates the recognition accuracy and exploration of emotional facial expressions

**DOI:** 10.3389/fnbeh.2014.00255

**Published:** 2014-07-23

**Authors:** Sabrina Boll, Matthias Gamer

**Affiliations:** Department of Systems Neuroscience, University Medical Center Hamburg-EppendorfHamburg, Germany

**Keywords:** 5-HTTLPR, eye gaze, amygdala, social perception, facial emotions

## Abstract

Individual genetic differences in the serotonin transporter-linked polymorphic region (5-HTTLPR) have been associated with variations in the sensitivity to social and emotional cues as well as altered amygdala reactivity to facial expressions of emotion. Amygdala activation has further been shown to trigger gaze changes toward diagnostically relevant facial features. The current study examined whether altered socio-emotional reactivity in variants of the 5-HTTLPR promoter polymorphism reflects individual differences in attending to diagnostic features of facial expressions. For this purpose, visual exploration of emotional facial expressions was compared between a low (*n* = 39) and a high (*n* = 40) 5-HTT expressing group of healthy human volunteers in an eye tracking paradigm. Emotional faces were presented while manipulating the initial fixation such that saccadic changes toward the eyes and toward the mouth could be identified. We found that the low vs. the high 5-HTT group demonstrated greater accuracy with regard to emotion classifications, particularly when faces were presented for a longer duration. No group differences in gaze orientation toward diagnostic facial features could be observed. However, participants in the low 5-HTT group exhibited more and faster fixation changes for certain emotions when faces were presented for a longer duration and overall face fixation times were reduced for this genotype group. These results suggest that the 5-HTT gene influences social perception by modulating the general vigilance to social cues rather than selectively affecting the pre-attentive detection of diagnostic facial features.

## Introduction

Numerous studies have investigated how a functional genetic variation within the promotor region of the serotonin transporter gene (*SLC6A4*, serotonin transporter-linked polymorphic region [5-HTTLPR]) modulates the processing of emotional stimuli and the susceptibility to mood and anxiety disorders (Lesch et al., 1996; Hariri et al., 2002; Kenna et al., 2012). The polymorphism influences the transcriptional efficacy of the serotonin transporter gene and is comprised of a low-expressing short (s-allele) and a high-expressing long (l-allele) variant (Lesch et al., 1996; Greenberg et al., 1999). A relatively consistent finding is the increased emotional and stress sensitivity of s-allele carriers (Hariri and Holmes, 2006; Canli and Lesch, 2007; Caspi et al., 2010). In summary, s-allele carriers demonstrated exaggerated amygdala responses to emotional faces in previous studies (Hariri et al., 2002; Munafò et al., 2008), as well as increased startle responses (Brocke et al., 2006), a stronger attentional bias for negatively valenced words and other threat-related stimuli (Beevers et al., 2007; Kwang et al., 2010; Pérez-Edgar et al., 2010; Carlson et al., 2012; Pergamin-Hight et al., 2012), but also greater difficulty disengaging their attention from both happy and sad stimuli (Beevers et al., 2009, 2010, 2011), and a higher sensitivity to happy than to sad faces (Koizumi et al., 2013). Taken together, the 5-HTTLPR is currently thought to affect behavior in a for-better-or-for-worse manner by modulating sensitivity thresholds to emotional cues via increased reactivity in corticolimbic brain structures (Belsky et al., 2009; Homberg and Lesch, 2011). In this context, the serotonin transporter gene variation has also been discussed to play an important role in social perception, presumably by affecting the sensitivity to social factors (Canli and Lesch, 2007; Kiser et al., 2012). However, the functional and behavioral implications of the genetically driven differences in the sensitivity to socio-emotional cues and amygdala reactivity for social perception have rarely been investigated and results from previous studies are often restricted to special populations (Marsh et al., 2006; Jacobs et al., 2011; Székely et al., 2011; Koizumi et al., 2013).

Another line of research demonstrated that amygdala activation in response to facial emotions is functionally implicated in directing spatial attention toward diagnostically relevant facial features (Gamer and Büchel, 2009; Gamer et al., 2010; Kliemann et al., 2012; Scheller et al., 2012). In these studies, faces were unpredictably shifted upward or downward on each trial such that participants initially fixated either on the eye or mouth region. Eye tracking data indicates that participants orient their gaze more frequently toward the eyes, but this gaze bias was attenuated when the mouth region was of greater diagnostic relevance (e.g., for happy faces). At the neural level, this gaze orientation behavior was accompanied by amygdala activation and an enhanced functional coupling between the amygdala and the superior colliculi (Gamer and Büchel, 2009; Gamer et al., 2010).

Here, we examined whether the serotonin transporter gene variation influences social perception by modulating the exploration of facial expressions of emotion. Due to the genetically driven variations in emotionality and amygdala activation, we expected to find differences between participants carrying the low-expressing 5-HTTLPR variant (low 5-HTT) and volunteers endowed with the 5-HTTLPR long variant (high 5-HTT) in several face scanning parameters extracted from eye tracking data. We predicted that the low vs. the high 5-HTT group would more frequently direct their attention toward socially relevant facial features and show a generally increased vigilance to facial cues as indicated by faster fixation changes and more intense face scanning (reduced fixation times and more saccades).

## Methods and materials

### Sample

Eighty-three Caucasian participants were initially selected from a large pool of subjects according to the 5-HTTLPR and the a/g single nucleotide polymorphism rs25531 of the 5-HTTLPR (Hu et al., 2006). Four subjects were excluded from further analyses—one subject due to insufficient task performance and three participants because of poor data quality (less than 65% valid eye tracking trials). In the final sample, the low 5-HTT expressing group [ss (*n* = 21), s/lg (*n* = 15), lg/lg (*n* = 3)] consisted of 39 volunteers (mean age 26.08, age range 21–36 years; 16 females), whereas the high 5-HTT expressing group (la/la) included a total of 40 participants (mean age 26.30, age range 19–39 years; 15 females). To increase statistical power and maximize differences between the genotype groups, volunteers belonging to the medium 5-HTT expressing group (la/lg, s/la) were not recruited. Participants in both groups had a comparable level of education. Most of them were students (77.50% in the high 5-HTT group and 74.36% in the low 5-HTT group), the others were either employees or freelancers (20.00% in the high 5-HTT group and 12.82% in the low 5-HTT group) and the remaining participants reported another type of employment.

Volunteers completed German versions of the Beck Depression Inventory (BDI; Hautzinger et al., 1994), the State-Trait Anxiety Inventory (STAI-T; Spielberger et al., 1983), the Positive and Negative Affect Schedule (PANAS; Watson et al., 1988), the Social Desirability Scale (SDS-17; Stöber, 2001), the Social Interaction Anxiety Scale (SIAS; Mattick and Clarke, 1998), two Alexithymia Questionnaires (BVAQ; Vorst and Bermond, 2001; TAS-20; Bagby et al., 1994) and the Reading Mind in the Eyes Test (RMET; Baron-Cohen et al., 2001). As shown in Table 1, volunteers in the two genotype groups did not differ significantly with respect to questionnaire values (all *p*-values > 0.05). The study was approved by the Ethics Committee of the Medical Board in Hamburg, Germany, and written informed consent was obtained from all subjects prior to the examination.

**Table 1:** **Questionnaire values for each subscale separated for the high and the low 5-HTT group**.

**Questionnaire**	**Subscales**	***M* (*SD*)**		**Range**		**Group comparisons**	
		**High 5-HTT**	**Low 5-HTT**	**High 5-HTT**	**Low 5-HTT**	***t*-values**	***p*-values**
BDI		4.50 (4.16)	3.28 (3.35)	0–17	0–14	1.43	0.16
PANAS	Negative affect	17.60 (5.05)	17.28 (4.90)	10–31	10–35	0.28	0.77
	Positive affect	33.95 (5.04)	35.15 (4.20)	19–45	27–43	1.15	0.25
STAI-T		33.88 (6.34)	33.97 (6.02)	22–51	25–48	0.07	0.94
TAS-20	Difficulty describing feelings	11.95 (4.38)	11.95 (4.07)	7–22	7–24	0.00	0.99
	Difficulty identifying feelings	12.13 (4.71)	11.97 (4.46)	5–22	5–22	0.15	0.88
	Externally-oriented thinking	16.68 (3.80)	17.26 (5.13)	9–27	8–31	0.57	0.57
	Total	40.75 (10.72)	41.18 (10.57)	21–66	23–62	0.18	0.93
BVAQ	Poor insight	21.35 (7.14)	21.28 (6.27)	8–36	11–34	0.04	0.96
	Poor verbalizing	21.93 (5.75)	21.62 (5.44)	11–34	9–33	0.25	0.81
	Poor analysing	18.03 (4.67)	18.08 (5.22)	8–30	9–30	0.05	0.96
	Poor fantasizing	21.08 (5.69)	20.72 (5.45)	10–35	10–35	0.28	0.78
	Poor emotional excitability	17.42 (5.17)	17.75 (5.41)	9–28	8–34	0.27	0.79
	Total	99.80 (20.87)	99.43 (17.92)	55–140	55–142	0.08	0.94
SES-17		10.73 (2.39)	10.69 (2.95)	7–15	4–16	0.05	0.96
SIAS		15.43 (8.51)	14.38 (7.14)	1–35	2–28	0.59	0.56
RMET		25.88 (3.74)	26.72 (3.09)	12–33	21–32	1.09	0.28

*BDI, Becks Depression Inventory; PANAS, Positive and Negative Affect Schedule; STAI-T, State-Trait Anxiety Inventory-Trait Version; TAS, Toronto Alexithymia Scale; BVAQ, Bermond and Vorst Alexithymia Questionnaire; SES-17, Social Desirability Scale; SIAS, Social Interaction Anxiety Scale; RMET, Reading Mind in the Eyes Test*.

### Stimuli and procedure

The study was based on a 2 × 2 × 4 × 2 design with the between subjects factor genotype (high 5-HTT vs. low 5-HTT) and the within-subject factors initial fixation (eyes vs. mouth), emotional expression (angry, fearful, happy, neutral) and presentation time (150 vs. 2000 ms). Emotional faces were selected from established picture sets including the Karolinska directed Emotional Faces database (KDEF, http://www.emotionlab.se/resources/kdef), the NimStim Face Stimulus Set (http://www.macbrain.org/), Pictures of facial affect (Ekman and Friesen, 1971) and the FACES database (Ebner et al., 2010). These faces were manipulated to optimally fit the experimental requirements: firstly, differences in the height of both eyes were adjusted by slightly rotating the faces. Secondly, colored images were converted to grayscale and cropped with an ellipse to hide hair and ears of the faces and finally, the cumulative brightness was normalized across all images. 80 different male or female faces (5 trials per condition) were presented in random order during the course of the experiment.

**Figure 1:**
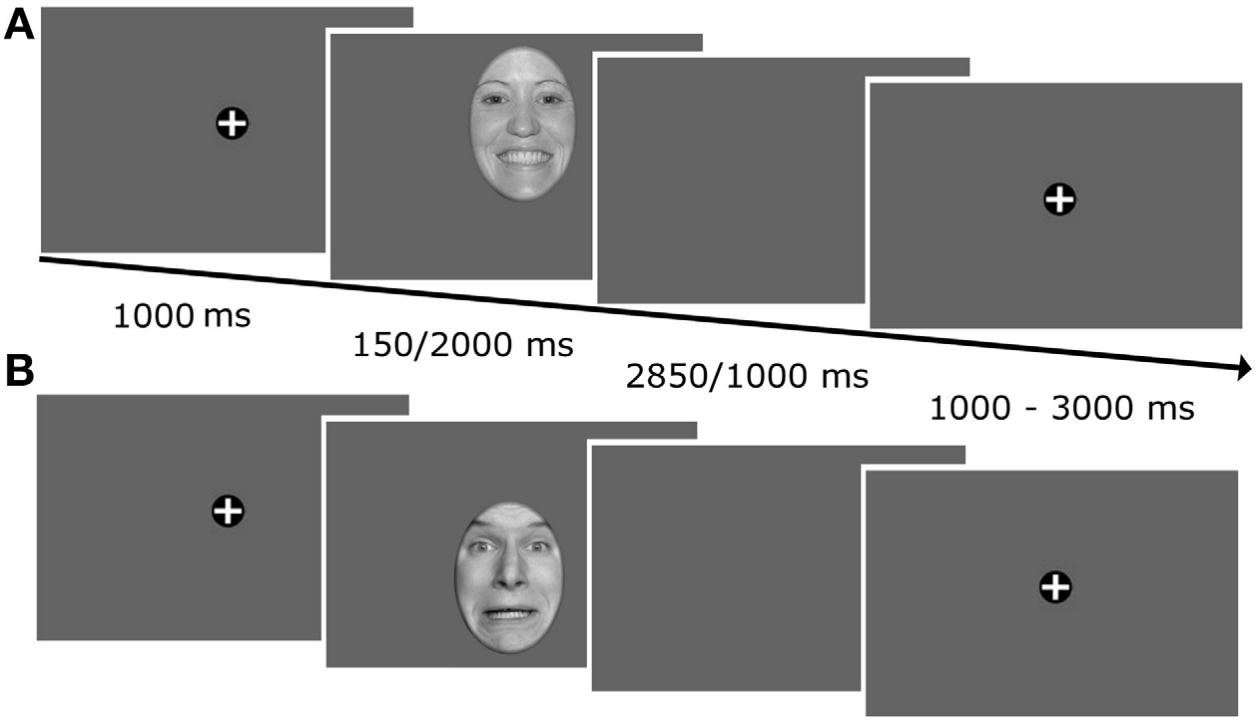
**Illustration of the trial timeline**. Faces were unpredictably shifted upwards or downwards on each trial such that either the mouth **(A)** or the eyes **(B)** were presented at fixation.

The task was to classify emotional expressions by pressing the corresponding key on a computer keyboard with the index and middle finger of both hands. During each trial, a fixation cross was presented for 1000 ms followed by a face which was presented for 150 or 2000 ms, respectively (Figure 1). When the picture had disappeared, a blank gray screen was presented for 2850 or 1000 ms to achieve an overall trial length of 3000 ms. During the randomly varying intertrial interval (1000–3000 ms) a fixation cross was shown on the screen. For each face presentation, the initial fixation was manipulated by unpredictably shifting the faces either upwards or downwards such that either the mouth or the eye region was fixated first. Participants were instructed to fixate the fixation cross, whenever it was presented on the screen, to look wherever they wanted to, when the faces or the subsequent empty screen were presented, and to avoid blinks during this time period. In addition, they were asked to answer as quickly and accurately as possible.

### Data acquisition

Eye movements were monitored using a remote infrared pupil-corneal reflection-based eye tracking system (Eye-Link 1000, SR Research Ltd., Ottawa, Canada) with a sampling rate of 1000 Hz. The head location was fixed using a chin rest and a forehead bar. Before the start of the experiment, the eye tracking camera was adjusted and a nine point calibration procedure was applied. Task presentation and recording of behavioral responses were performed with the software Presentation (Neurobehavioral Systems, Albany, CA, USA). Stimuli were presented on a 20” Samsung SyncMaster 204B display (40.64 × 30.48 cm) with a resolution of 1600 by 1200 pixels and a refresh rate of 60 Hz. The viewing distance to the monitor was 55 cm.

### Data analysis

#### Behavioral data

Correct responses (proportion of hits) and reaction times were analyzed using a 2 × 2 × 4 × 2 repeated measures ANOVA with the between subjects factor genotype and the within subject factors initial fixation, emotion and presentation time. Reaction times were only analyzed for trials in which a correct answer was given and corrected for outliers by removing trials in which the response time exceeded 3000 ms (0.72% of all trials across subjects). One subject was excluded from this analysis due to missing data. Behavioral data were also analyzed separately for the short and for the long stimulus duration using a 2 × 2 × 4 repeated measures ANOVA with the factors genotype, initial fixation and emotion.

#### Eye movement data

For all eye movement measures, trials which included fixation changes with an amplitude of more than 1° or blinks during a baseline period of −300 to 150 ms relative to the onset of the stimulus were excluded from further analysis. Similarly, trials were excluded when the blink-free time period amounted to less than 85% of the whole trial.

To determine whether the serotonin transporter gene variation affects gaze orientation toward diagnostically relevant facial features, we extracted the first (reflexive) saccade after stimulus onset in the vertical direction. These initial fixation changes were detected, when they occurred within a time interval of 150–1000 ms after stimulus onset and their amplitude exceeded 1°. When the eyes were initially fixated, downward fixation changes toward the mouth were identified, whereas upward fixation changes toward the eyes were scored when the mouth was presented first. The number of first saccades was then divided by the number of valid trials in the corresponding experimental condition. A 2 × 2 × 4 × 2 repeated measures ANOVA with the between subjects factor genotype and the within subject factors initial fixation, emotion and presentation time was calculated to determine whether the proportion of fixation changes varied as a function of the experimental conditions. Subsequently, separate 2 × 2 × 4 repeated measures ANOVA with the factors genotype, initial fixation and emotion were conducted for the short and the long presentation time.

Analyses of the eye tracking data further aimed at identifying differences in the vigilance and intensity of face scanning between experimental conditions and groups. The latency of fixation changes was therefore identified as an additional eye tracking measure. To avoid the exclusion of numerous participants because of empty cells (i.e., missing fixation changes in specific experimental conditions), latencies were averaged across all conditions and compared between the two genotype groups using a two-sample *t*-test.

We further investigated how thoroughly participants scanned the faces during the long presentation time by extracting two additional measures from the eye tracking data. First, we analyzed the mean number of all saccades in either direction occurring between 150 and 2000 ms after stimulus onset as a function of the experimental manipulations. Again, saccades <1° were excluded from further analysis. Second, total dwell times were determined for the 2000 ms stimulus duration. We identified the amount of time subjects spent looking at predefined regions of interests for the face area and divided these values by the total stimulus duration of 2000 ms. For both measures, we calculated a 2 × 2 × 4 repeated measures ANOVA with the between subjects factor genotype and the within subject factors initial fixation and emotion to investigate effects of the experimental manipulations on these face scanning parameters.

Statistical analyses were performed using the statistical programming language R (www.r-project.org). A significance threshold of *p* < 0.05 was applied to all statistical tests. Partial Cohen's f is reported as an effect size index (Cohen, 1988).

## Results

### Behavioral data

#### Proportion of correct responses

**Figure 2:**
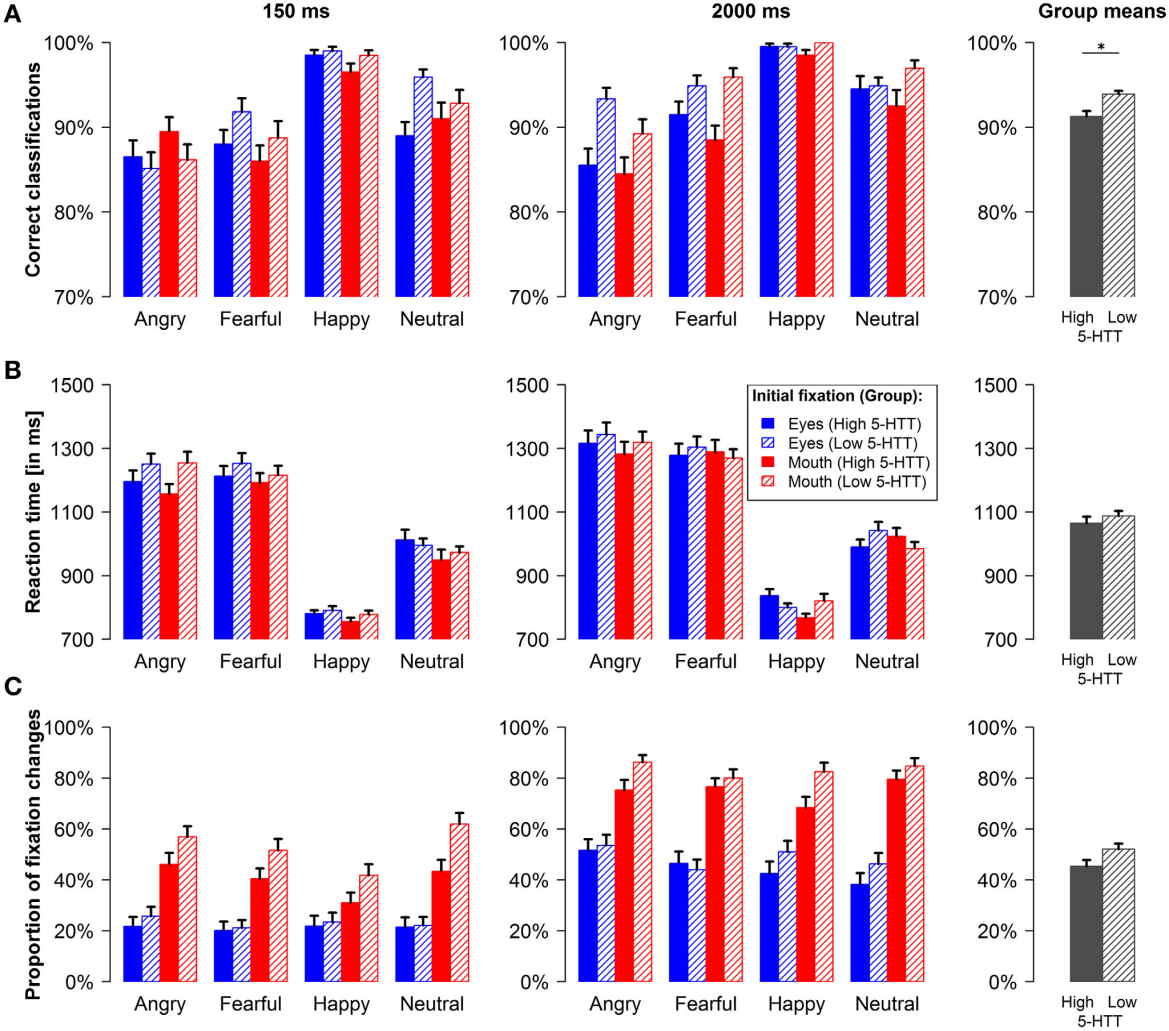
**Behavioral data and fixation changes**. Proportion of correct emotion classifications **(A)**, reaction times **(B)** and proportion of reflexive upward and downward fixation changes **(C)** for the short and the long presentation time are shown for every condition and pooled across conditions for the two genotype groups (group means, rightmost column). Error bars indicate s.e.m. ^*^p < 0.05.

Volunteers in the low 5-HTT group were more accurate in classifying emotional expressions as compared to participants in the high 5-HTT group [main effect genotype: *F*_(1, 77)_ = 6.00, *p* < 0.05, *f* = 0.28; see group means in Figure 2A, rightmost column]. In both groups, more correct responses were observed during the 2000 ms relative to the 150 ms stimulus duration [main effect presentation time: *F*_(1, 77)_ = 18.40, *p* < 0.001, *f* = 0.49]. Moreover, the largest hit rates were observed for happy faces and the lowest for angry faces [main effect emotion: *F*_(3, 75)_ = 42.59, *p* < 0.001, *f* = 0.60]. Neither the main effect of the initial fixation nor any of the interaction effects reached statistical significance. When calculating separate ANOVAs for both presentation times, we observed a significant main effect of the factor emotion during the short [*F*_(3, 75)_ = 29.12, *p* < 0.001, *f* = 0.47] as well as during the long stimulus duration [*F*_(3, 75)_ = 26.58, *p* < 0.001, *f* = 0.52]. However, the genotype effect was only present, when stimuli were presented for 2000 ms [*F*_(1, 77)_ = 8.37, *p* < 0.01, *f* = 0.33].

#### Response latencies

Participants were slower in pressing the correct button during the 2000 ms stimulus duration [main effect presentation time: *F*_(1, 77)_ = 29.97, *p* < 0.001, *f* = 0.62] as can be seen in Figure 2B. Additionally, volunteers responded faster, when the mouth and not the eye region was initially fixated [main effect initial fixation: *F*_(1, 77)_ = 4.91, *p* < 0.05, *f* = 0.25]. The fastest reactions were observed for happy faces, whereas angry and fearful faces resulted in the slowest responses [main effect emotion: *F*_(3, 75)_ = 125.54, *p* < 0.001, *f* = 1.53]. In addition, we obtained a significant 4-way interaction involving all experimental factors [*F*_(3, 75)_ = 4.14, *p* < 0.01, *f* = 0.14] and the interaction of presentation time by emotion [*F*_(3, 75)_ = 4.39, *p* < 0.05, *f* = 0.22] was also significant.

To further characterize these effects, we calculated separate ANOVAs for the short and the long stimulus duration. During the long presentation time, a significant 3-way interaction of genotype, initial fixation and emotion was observed [*F*_(3, 75)_ = 4.58, *p* < 0.01, *f* = 0.16]. Figure 2B (right panel) suggests that participants in the high 5-HTT group reacted faster during the long stimulus duration, when facial features of higher diagnostic relevance (e.g., the eyes of fearful and neutral faces and the mouth of happy faces) were presented at fixation, whereas the opposite applied for the low 5-HTT group. For both presentation times, the main effect of emotion was significant [150 ms duration: *F*_(3, 75)_ = 111.74, *p* < 0.001, *f* = 1.26; 2000 ms duration: *F*_(3, 75)_ = 111.90, *p* < 0.001, *f* = 1.44]. All other effects in the full and in the reduced models did not reach statistical significance.

### Eye movement data

The average percentage of valid trials was 90.17% (range = 72.50–100%). No differences between the two groups could be observed (low 5-HTT: *M* ± *SD* = 89.78 ± 8.58%; high 5-HTT: *M* ± *SD* = 90.56 ± 6.91%, *p*-value > 0.6).

#### Proportion of fixation changes

Figure 2C depicts the proportions of reflexive fixation changes as an indicator for gaze orientation toward diagnostically relevant facial features. Overall, participants made significantly fewer saccades when the eye and not the mouth region was initially fixated [main effect initial fixation: *F*_(1, 77)_ = 36.92, *p* < 0.001, *f* = 0.69]. In accordance with previous findings (Gamer and Büchel, 2009; Gamer et al., 2010; Scheller et al., 2012), reflexive gaze changes further varied according to the distribution of diagnostically relevant features in the face [interaction of emotion and initial fixation: *F*_(3, 75)_ = 11.69, *p* < 0.001, *f* = 0.36]. Thus, saccades toward the eyes were more likely for fearful, angry, and neutral faces, whereas happy faces did less frequently trigger gaze changes when the mouth was initially fixated. Overall, happy faces evoked fewer saccades than all other emotional expressions and the largest proportion of gaze changes was observed for angry faces [main effect emotion: *F*_(3, 75)_ = 8.89, *p* < 0.001, *f* = 0.69]. All participants made more fixation changes during the long presentation time [main effect presentation time: *F*_(1, 77)_ = 180.87, *p* < 0.001, *f* = 1.53] and the proportion of saccades toward the eyes relative to saccades toward the mouth was increased for the long stimulus duration [interaction of presentation time and initial fixation: *F*_(1, 77)_ = 4.36, *p* < 0.05, *f* = 0.24]. No statistical evidence for differences between the two genotype groups was found.

Separate ANOVAs for the short and the long stimulus duration revealed similar effects for both conditions. Again, a main effect of the factor emotion [short stimulus presentation: *F*_(3, 75)_ = 5.65, *p* < 0.01, *f* = 0.33; long stimulus presentation: *F*_(3, 75)_ = 4.36, *p* < 0.01, *f* = 0.22] as well as a main effect of the initial fixation position [short stimulus presentation: *F*_(1, 77)_ = 21.13, *p* < 0.001, *f* = 0.53; long stimulus presentation: *F*_(1, 77)_ = 45.66, *p* < 0.001, *f* = 0.77] was observed. The interaction of emotion and initial fixation was also significant for both presentation times [short stimulus presentation: *F*_(3, 75)_ = 7.56, *p* < 0.001, *f* = 0.32; long stimulus presentation: *F*_(3, 75)_ = 5.53, *p* < 0.01, *f* = 0.24].

#### Latency of fixation changes

The latency of fixation changes differed significantly between the two genotype groups [*t*_(77)_ = 2.02, *p* < 0.05]. Participants in the low 5-HTT group made faster fixation changes (*M* = 333.48 ms, *SD* = 69.69 ms) than volunteers in the high 5-HTT group (*M* = 382.78 ms, *SD* = 119.82 ms).

#### Mean number of all saccades

**Figure 3:**
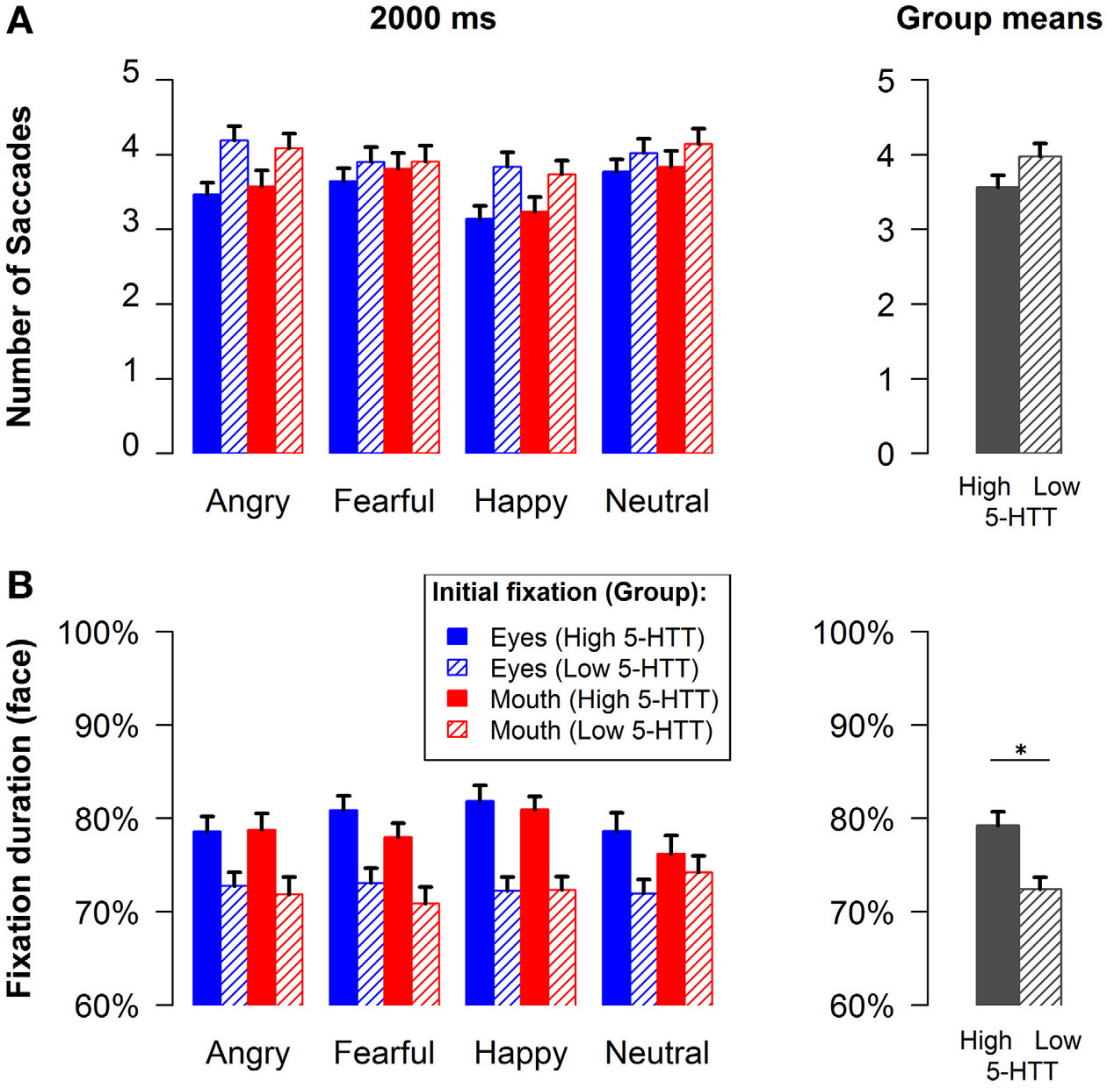
**Mean number of all fixation changes (A) and face fixation duration (B) for the long stimulus presentation time**. Group means are represented by the gray bars in the rightmost column. Error bars indicate s.e.m.^*^p < 0.05.

Analyses of the mean number of all saccades in either direction during the long presentation time revealed a significant interaction of genotype by emotion [*F*_(3, 75)_ = 3.91, *p* < 0.05, *f* = 0.19, see Figure 3A]. Thus, participants in the low 5-HTT as compared to the high 5-HTT group made more saccades, particularly when considering angry and happy faces. Additionally, less saccades were observed for happy faces relative to all other emotions across all participants [main effect emotion: *F*_(3, 75)_ = 7.18, *p* < 0.001, *f* = 0.33].

#### Face fixation duration

Figure 3B illustrates the amount of time subjects fixated the faces relative to the presentation time of 2000 ms. The group means in the lower right panel of the figure show that participants in the high 5-HTT group had longer fixation times as compared to the low 5-HTT group [main effect genotype: *F*_(1, 77)_ = 6.08, *p* < 0.05, *f* = 0.28]. All other effects failed to reach statistical significance. It can be seen that the fixation duration measure virtually mirrors the mean number of saccades. However, it is probably more sensitive to small effects than the number of saccades as it is represented at a continuous scale.

## Discussion

In this study, we investigated effects of the allelic variation in the serotonin transporter gene on emotion recognition and simultaneous gaze orienting. Volunteers with a low-expression form of the 5-HTTLPR were significantly more accurate in classifying emotional facial expressions relative to volunteers with the high-expression variant. As separate analyses for both presentation times demonstrated, this effect seemed to be mainly driven by emotion classification during the long stimulus duration. The improved accuracy in the low 5-HTT group was accompanied by shorter saccadic latencies and reduced face fixation times as well as an enhanced number of saccades for certain emotions in the low 5-HTT group, when faces were presented for 2000 ms. The observed pattern of results presumably reflects an increased vigilance toward social cues in carriers of the low 5-HTT expressing variant and a tendency for more intense face scanning. No significant effect of 5-HTTLPR on the interaction of emotion and initial fixation was observed for the proportion of fixation changes indicating that gaze orientation toward diagnostic facial features was not affected by the allelic variation of the 5-HTT gene.

Previous studies highlighted the increased autonomic and neuronal reactivity of s-allele carriers to socio-emotional cues such as facial expressions (Hariri et al., 2002; Brocke et al., 2006). In the current study, the low 5-HTT group was significantly better in classifying facial expressions as compared to the high 5-HTT group irrespective of the type of emotion, particularly when faces were presented for 2000 ms. Our results therefore extend previous findings by demonstrating a behavioral advantage for carriers of the low-functioning 5-HTTLPR allele in the recognition of facial expressions which act as integral signals for social interaction. The current findings are therefore consistent with recent claims suggesting that the 5-HTT gene plays an important role in shaping social behavior and perception (Canli and Lesch, 2007; Kiser et al., 2012). The 5-HTTLPR short variant has been associated with low scores on the personality trait “agreeableness” (Lesch et al., 1996), increased social blushing (Domschke et al., 2009) and increased responsiveness to social support ameliorating the risk for depression (Kaufman et al., 2004).

Previous studies have also directly investigated the association between the 5-HTTLPR and emotion recognition and have obtained mixed results. For instance, Székely et al. (2011) demonstrated that children homozygous for the s allele were less accurate in recognizing fearful faces than children carrying at least one l allele in an emotion-matching task. Another study with adolescent participants found that s allele carriers whose mothers reported a depressive history made more errors in identifying emotional faces than adolescents with an intermediate or high expressing genotype, with or without a history of adversity (Jacobs et al., 2011). Moreover, Marsh et al. (2006) demonstrated in a sample of 26 participants that tryptophan depletion significantly impaired the recognition of fearful facial expressions in s carriers but not ll homozygotes. Others studies reported emotion-specific effects, showing for instance that s allele carriers had a reduced bias to perceive neutral faces as expressions of happiness (Gohier et al., 2014) and an impaired recognition of happy but better recognition of fearful faces (Defrancesco et al., 2011).

In the current study, we observed improved emotion recognition for all types of emotions in the low as compared to the high 5-HTT group rather than a specific impairment in these subjects for fearful faces that was found in the above mentioned studies. One reason for this inconsistency could be that previous studies were based on specific populations and are hardly comparable to the current results with respect to the exact genotype grouping and the applied emotion recognition tasks. Moreover, our findings are in line with the frequently observed increased autonomic and amygdala reactivity of the low as compared to the high expressing 5-HTTLPR variant (Munafò et al., 2008). Nevertheless, a replication of the current results on emotion recognition in a larger sample would strengthen our findings as recent meta-analyses have generally criticized research on candidate genes for low power and possible publication bias in replication attempts (Duncan and Keller, 2011).

Apart from emotion recognition accuracy, attentional measures were assessed here and the current study is the first to evaluate gaze preferences in 5-HTT genotypes with respect to facial features in a controlled eye-tracking paradigm. Previously, a study in macaques reported a gaze preference for the eye region in a monkey endowed with the short s-allele of the 5-HTT gene (Gibboni et al., 2009). In contrast, Watson et al. (2009) reported reduced fixation of the eye region along with increased sympathetic responses to images of high-status males in carriers of the rh5-HTTLPR s-variant. In humans, the link between gaze orientation and the 5-HTT gene has been studied in free-viewing conditions suggesting a gaze preference for positive stimuli in a low relative to a high 5-HTT group (Beevers et al., 2011). Here, we replicated earlier findings showing that participants orient their gaze toward diagnostically relevant facial features irrespective of task demands (Gamer and Büchel, 2009; Gamer et al., 2010; Kliemann et al., 2012; Scheller et al., 2012). Across both genotype groups, participants gazed more often to the eyes of the emotional faces. This gaze preference for the eye region was however reduced for happy faces. The factor genotype did not modulate this interaction of initial fixation and emotion. Against our initial hypothesis, our data therefore provide no evidence for a stronger gaze preference for diagnostic facial features in the low 5-HTT group. Yet, analysis of the response latency data revealed the expected interaction of genotype, emotion and initial fixation for the long stimulus duration. Participants in the high 5-HTT group responded faster, when diagnostically relevant facial features (such as the eyes of fearful and neutral faces and the mouth of happy faces) were presented at fixation. Volunteers in the low 5-HTT group in contrast, showed the opposite response pattern and had shorter response latencies in trials in which fixation changes toward the other facial features of higher diagnostic relevance were beneficial (e.g., when the mouth of fearful faces was presented at fixation). However, as the effect observed for reaction time data does not match the results obtained for the proportion of fixation changes, it does not provide sufficient evidence for a genetic modulation of the reflexive attentional orienting toward diagnostic facial features.

Considering the group differences observed for the other face scanning parameters in the current study, it is more likely that the 5-HTT gene influences social perception and recognition accuracy in a more general way (i.e., by influencing vigilance to social cues) in line with current conceptualizations of the 5-HTTTLPR as a vigilance-modulating polymorphism (Homberg and Lesch, 2011). Earlier studies particularly accentuated deficits associated with the 5-HTTLPR short variant by demonstrating for instance increased fear-potentiated startle responses (Brocke et al., 2006), exaggerated amygdala activity in response to fearful faces (Hariri et al., 2002; Munafò et al., 2008) as well as an increased risk for depression and anxiety disorders in interaction with environmental factors (Lesch et al., 1996; Kenna et al., 2012). More recent reports however, have suggested a conceptual change based on findings which indicate that the s allele actually increases sensitivity not only to negative but also to positive stimuli pointing to a more general role of the 5-HTTLPR short variant in influencing environmental sensitivity (Uher, 2009; Homberg and Lesch, 2011). Fox and colleagues, for instance, reported that a low vs. a high 5-HTT group developed stronger biases for positive and negative affective pictures (Fox et al., 2011). Moreover, s-allele carriers had greater difficulty disengaging attention from both happy and sad faces (Beevers et al., 2009, 2010) and outperformed carriers of the long allelic form in a variety of cognitive tasks (Homberg and Lesch, 2011). Thus, growing evidence suggests that the serotonin transporter gene variation can be conceptualized as a plasticity rather than a vulnerability gene, which alters sensitivity thresholds to environmental stimuli by modulating the level of vigilance (Belsky et al., 2009; Homberg and Lesch, 2011). At the neural level, the genetically-driven hypervigilance in s-allele carriers is presumably mediated by hyperactivity in corticolimbic structures, such as the amygdala. Alternatively, a reduced ability to regulate the impact of aversive stimuli may underlie hypervigilance in s-allele carriers as reduced BOLD responses to fearful vs. neutral faces in l- but not in s-allele carriers have been reported in the subgenual cingulate cortex, for example, whereas no differences in amygdala activity could be found in this study (O'Nions et al., 2011).

The idea of hypervigilance in s-allele carriers fits well with our finding of shorter saccadic onset times in the low relative to the high 5-HTT group. Moreover, face fixation times were reduced for the low 5-HTT group during the long stimulus presentation time and we observed an interaction between emotion and genotype for the mean number of saccades during the long stimulus presentation time. These results suggest a hypervigilant gaze orientation and more intense face scanning in the low 5-HTT group relative to carriers of two l-alleles which might relate to the increased emotion recognition rates in the low 5-HTT group. Interestingly, group differences in the mean number of saccades were most pronounced for happy and angry faces. On the one hand, the number of saccades was reduced for happy faces in the high 5-HTT group relative to all other emotions, whereas the low 5-HTT group did not show this reduction. As happy faces produced the highest hit rates and the lowest reaction times across both groups, this finding underlines a hypervigilant gaze sampling behavior in volunteers carrying the low 5-HTT expressing variant, whereas participants in the high 5-HTT group seem to focus more strongly on relevant information. On the other hand, participants in the low 5-HTT group made slightly more saccades for angry faces relative to the other emotions and again this was not the case for the other genotype group. As previous studies reported a threat-related attentional bias in participants endowed with a low-expressing variant of the 5-HTT gene (Beevers et al., 2007; Kwang et al., 2010; Pérez-Edgar et al., 2010; Carlson et al., 2012; Pergamin-Hight et al., 2012), the relatively high number of saccades in the low 5-HTT group observed in the current study can be understood as enhanced gaze sampling in response to most threatening conditions.

Taken together, the low 5-HTT group in our study made more correct emotion classifications particularly during the long presentation time, faster fixation changes and showed enhanced gaze sampling behavior (again during the long duration face presentation) as indicated by reduced face fixation times and more saccades, particularly for happy and angry faces. We assume that this pattern of results reflects enhanced vigilance to social cues in the low 5-HTT group. Thus, our data support the connotation of the serotonin transporter gene as a vigilance-modulating gene with hypervigilance in carriers of the low 5-HTT expressing group possibly leading to benefits (improved emotion recognition) in the current task. The assumption that 5-HTTLPR particularly modulates attentional orienting to diagnostic criteria of emotional faces could not be supported by the current data.

### Conflict of interest statement

The authors declare that the research was conducted in the absence of any commercial or financial relationships that could be construed as a potential conflict of interest.
